# Coral life history and symbiosis: Functional genomic resources for two reef building Caribbean corals, *Acropora palmata *and *Montastraea faveolata*

**DOI:** 10.1186/1471-2164-9-97

**Published:** 2008-02-25

**Authors:** Jodi A Schwarz, Peter B Brokstein, Christian Voolstra, Astrid Y Terry, David J Miller, Alina M Szmant, Mary Alice Coffroth, Mónica Medina

**Affiliations:** 1 Biology Department, Vassar College, 124 Raymond Avenue, Poughkeepsie, NY 12604, USA; 2 Joint Genome Institute, Department of Energy, 2800 Mitchell Drive, Walnut Creek, CA 94598, USA; 3 School of Natural Sciences, PO Box 2039, University of California, Merced, CA 95344, USA; 4 Comparative Genomics Center, Molecular Sciences Building 21, James Cook University, Townsville, Queensland 4811, Australia; 5 Center for Marine Science, Center for Marine Science, 5600 Marvin K. Moss Lane, Wilmington, NC 28409 600 Marvin K. Moss Lane, Wilmington, NC 28409, USA; 6 Department of Geological Sciences, 661 Hochstetter Hall, State University of New York, Buffalo, NY 14260, USA

## Abstract

**Background:**

Scleractinian corals are the foundation of reef ecosystems in tropical marine environments. Their great success is due to interactions with endosymbiotic dinoflagellates (*Symbiodinium *spp.), with which they are obligately symbiotic. To develop a foundation for studying coral biology and coral symbiosis, we have constructed a set of cDNA libraries and generated and annotated ESTs from two species of corals, *Acropora palmata *and *Montastraea faveolata*.

**Results:**

We generated 14,588 (*Ap*) and 3,854 (*Mf*) high quality ESTs from five life history/symbiosis stages (spawned eggs, early-stage planula larvae, late-stage planula larvae either infected with symbionts or uninfected, and adult coral). The ESTs assembled into a set of primarily stage-specific clusters, producing 4,980 (*Ap*), and 1,732 (*Mf*) unigenes. The egg stage library, relative to the other developmental stages, was enriched in genes functioning in cell division and proliferation, transcription, signal transduction, and regulation of protein function. Fifteen unigenes were identified as candidate symbiosis-related genes as they were expressed in all libraries constructed from the symbiotic stages and were absent from all of the non symbiotic stages. These include several DNA interacting proteins, and one highly expressed unigene (containing 17 cDNAs) with no significant protein-coding region. A significant number of unigenes (25) encode potential pattern recognition receptors (lectins, scavenger receptors, and others), as well as genes that may function in signaling pathways involved in innate immune responses (toll-like signaling, NFkB p105, and MAP kinases). Comparison between the *A. palmata *and an *A. millepora *EST dataset identified ferritin as a highly expressed gene in both datasets that appears to be undergoing adaptive evolution. Five unigenes appear to be restricted to the Scleractinia, as they had no homology to any sequences in the nr databases nor to the non-scleractinian cnidarians *Nematostella vectensis *and *Hydra magnipapillata*.

**Conclusion:**

Partial sequencing of 5 cDNA libraries each for *A. palmata *and *M. faveolata *has produced a rich set of candidate genes (4,980 genes from *A. palmata*, and 1,732 genes from *M. faveolata*) that we can use as a starting point for examining the life history and symbiosis of these two species, as well as to further expand the dataset of cnidarian genes for comparative genomics and evolutionary studies.

## Background

### The role of coral-dinoflagellate symbioses in coral reef ecosystems

Coral reefs are among the most biodiverse ecosystems on Earth and play important roles in ocean biogeochemical cycles. In particular, scleractinian corals are keystone species in tropical marine environments where they create the substrate and three dimensional structure of the reef ecosystem, and also provide the majority of primary productivity within the reef. Corals' reef-building capability arises from an endosymbiotic relationship with photosynthetic dinoflagellate symbionts (*Symbiodinium *spp.). The symbionts typically live enclosed within membrane-bound "symbiosomes" in host cells of the gastroderm. Corals derive photosynthate to fuel much of their metabolic requirements, while symbionts derive inorganic compounds from the host to fuel photosynthesis and symbiont growth [1]. The presence of a photosynthesizing symbiont profoundly influences rates of growth, reproduction, and CaCO_3 _deposition of scleractinian corals. While the photosynthetic nature of the symbionts restricts symbiont-bearing corals to the well-lit surface waters, it permits the partners to live in otherwise inhospitable nutrient-poor regions of the world's oceans, and sets the stage for the formation of reefs in shallow tropical water [2].

Coral reef ecosystems are currently under severe pressure from both local (i.e., inputs of sediments and chemical pollutants, over fishing) and large-scale changes in the marine environment [3]. In particular anomalous temperature and/or high UV levels can prompt widespread coral "bleaching" events in which the symbiosis fails and symbionts are jettisoned from the host. To better respond to these threats, it is imperative that we better understand the initiation and regulation of the cnidarian-dinoflagellate symbiosis. In this manuscript we report the development of new genomic tools that will facilitate these discoveries in two Caribbean coral species, *Acropora palmata *and *Montastraea faveolata*.

### Establishment of the host-symbiont relationship

The vast majority of scleractinian corals are obligately symbiotic, making it surprising that few species exhibit maternal (vertical) transmission of symbionts to their offspring [4,5]. Instead, most corals produce offspring that lack symbionts, and these offspring are faced with the task of acquiring symbionts from the environment. Newly settled hard corals and gorgonians can acquire zooxanthellae within a week when maintained on the reef as well as when cultured in the laboratory [6]. In most cases so far examined, symbiotic cnidarians take up symbionts by phagocytosis, during which symbionts that enter the host's gastric cavity are internalized by phagocytic cells of the gastroderm [7-10]. Occasionally, *Symbiodinium *has also been observed in the ectoderm of larvae [9], and in some coral species, *Symbdiodinium *infects the new host during embryonic development, before gastrulation [11].

The pattern of horizontal transmission presents an experimental system with which to study the development of the symbiosis at the molecular level since it is possible to collect large quantities of coral gametes that can be experimentally infected with symbionts after fertilization. This makes it possible to both control the timing and conditions under which coral larvae take up their first complement of symbionts, and to sample through different life history and symbiotic stages of corals.

### Coral comparative developmental genomics

Corals are members of the phylum Cnidaria, a basal metazoan group that includes jellyfish, sea anemones, and hydrozoans. Relative to other metazoans, cnidarians are characterized by embryonic development of only two tissue layers (endoderm and ectoderm), a lack of complex organ systems, and the development of fewer than a dozen cell types. EST/genomic sequencing projects for the hydrozoan *Hydra magnipapillata*, the sea anemone *Nematostella vectensis*, and the Indo-Pacific scleractinian coral *Acropora millepora *have revealed that cnidarians possess homologs for many developmental genes that control axial specification of the bilaterian body plan [12-16]. The recent publication of the whole genome of *N. vectensis *has revealed that a large portion of the gene repertoire is shared between cnidarians and vertebrates whereas substantial gene loss has occurred in the ecdysozoan model organisms (i.e. *Drosophila melanogaster *and *Caenorhabdites elegans*) [12,16]. Given the genetic complexity of cnidarians, a comparative analysis of scleractinian and bilaterian genomes would greatly enhance our understanding of a diverse array of metazoan biological processes, for example symbiosis [17], and calcification and biomineralization [18-20].

### Onset of Symbiosis

One of our primary goals is to investigate the transcriptome of the symbiotic relationship from both the host and symbiont perspectives. A large scale analysis is necessary as there are several biological processes that are likely to be invoked to establish a stable partnership. Mechanisms of host-symbiont recognition likely involve interactions between molecules present on the surface of both the symbiont and host cells, for example carbohydrate-lectin interactions, as were recently described for the coral *Fungia scutaria *[21]. Concomitant with the phagocytic uptake of symbionts, the host will likely exhibit some flavor of innate immune response, and that response will likely be manipulated by the symbiont such that it remains viable within the host cell [22]. There is evidence that one mechanism by which *Symbiodinium *manipulates host response is by interfering with maturation of the phagosome [23-25] Second, upon entry, it is likely that the underlying structural organization of the host cell/gastrodermal tissue must be modified significantly, as the symbiont occupies most of the volume of the host cell. This would likely include alterations in lipids and protein components of the cell, features that were implicated in the mature symbiosis in the sea anemone *Anthopleura elegantissima *[17,26] Third, the presence of *Symbiodinium *is correlated with rapid calcification of the coral skeleton, and therefore processes related to skeletonization (Puverel et al. 2007) are likely to be induced or upregulated during the onset of symbiosis.

### Experimental approach

To gain a large-scale cellular and molecular perspective on the biology of coral-dinoflagellate symbiosis, we have developed a suite of cDNA libraries that encompass significant stages in coral life history and symbiotic stages, including unfertilized eggs, two stages of embryonic development, cohorts of larvae that are either symbiotic or non-symbiotic, and adult coral. Partial sequencing of these libraries has produced a rich set of unigenes, from which we can probe the underlying biology of the coral-dinoflagellate symbiosis via downstream genomic approaches (i.e. microarrays).

**Figure 1:**
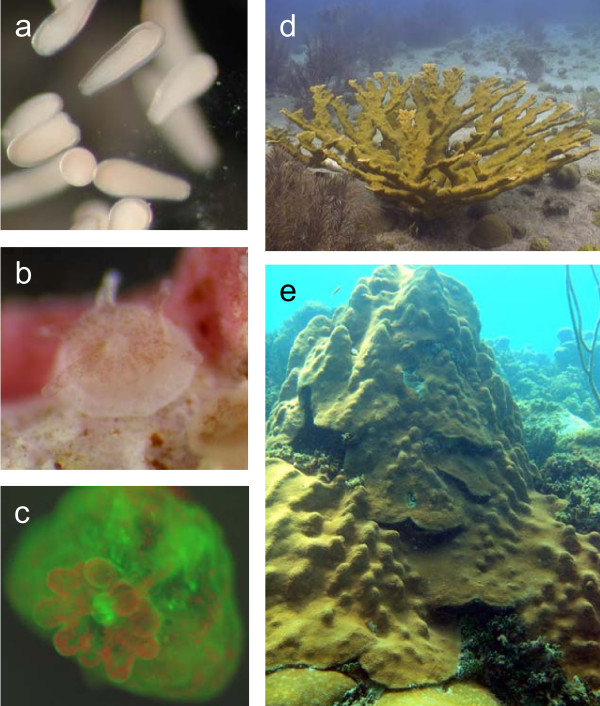
Life history stages of *A. palmata *and *M. faveolata*. **a, c, d **are *A. palmata*, **b, e **are *M. faveolata*. **a**) swimming planulae larvae, **b**) newly metamorphosed polyp containing brown symbionts in tentacles and oral disc, **c**) newly metamorphosed polyp photographed under blue light highlights the localization of symbionts primarily to the tentacles and oral disk of the host (red fluorescence is produced by chlorophyll within *Symbiodinium *and green fluorescence is a GFP-like protein produced by the coral, **d, e**) adult colonies of both coral species.

We targeted two scleractinian corals, *Montastraea faveolata *and *Acropora palmata *(Figure 1), both are major reef builders in the Caribbean. Over the past few dacades, the Caribbean has suffered massive declines in coral cover [27], and in May 2006 *A. palmata *was listed as a threatened species under the Endangered Species Act [28]. Both these coral species produce azooxanthellate eggs that can be fertilized and reared through the larval and post-metamorphic polyp stages to a stage that can be experimentally infected with selected strains of *Symbiodinium *available in culture. We generated ESTs from the earliest stages of the symbiosis, as well as later host-symbiont combinations.

In light of the deteriorating condition of coral reefs worldwide and the severity of coral bleaching events, it is critical that we utilize a large-scale approach to studying the onset, regulation, and maturation of symbiosis in scleractinian corals so that we can better understand the underlying biological processes involved.

## Results

### EST Assembly and Annotation

We sampled 5 life history/symbiosis stages for each species to make stage-specific cDNA libraries: spawned eggs (SE), early-stage planula larvae (EP), late-stage planula larvae that we either infected with symbionts (LPI) or left uninfected (LPU), and adult coral (AC). We generated 14,588 high quality ESTs for *A. palmata*, which assemble into 4,980 unigenes, and 3,854 high quality ESTs for *M. faveolata*, which assemble into 1,732 unigenes (Table 1). The ESTs are available through GenBank (accession# DR982333–DR988505, EY021828–EY031784, and FE038910–FE040597) and the unigene consensus sequences are available through the Marine Genomics Project website [29].

**Table 1:** Annotation of ESTs and unigenes (assembled EST clusters)

ESTs submitted to GenBank			
Library	Abbrev	*A. palmata*	*M. faveolata*
Spawned eggs^1^	SE	4018	201
Early planulae^2^	EP	2114	408
Late planulae (uninfected^2^	LPU	3111	1125
Late planulae (infected)	LPI	3323	559
Adult colony	AC	2022	1561

EST Unigene sets			

Total # cDNAs		8524	2273
# unigenes		4980	1732
Average # cDNAs/unigene		1.94	1.41

Unigene Blast annotations (1e-5)			

% nr blastx hits		31%	21%
% swissprot hits		25%	17%
% GO hits		26%	17%

Unigenes with no blast hits			

# encoding > 300 nt ORF		892	300
# encoding > 300 nt ORF with M		574	160
# ORFs + SignalP NN/HMM		97/111	22/28

^1^*A. palmata *96 hr and *M. faveolata *60 hr post fertilization

^2^*A. palmata *15 days and *M. faveolata *13 days post fertilization

**Figure 2:**
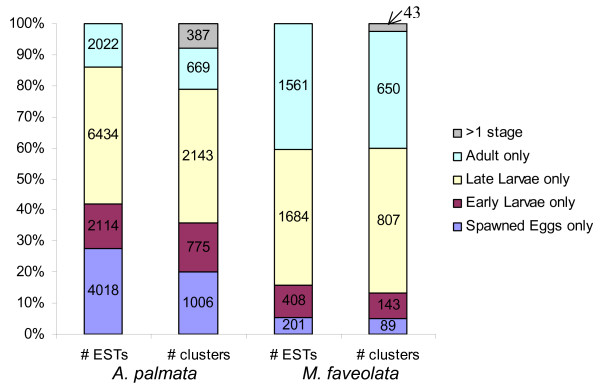
ESTs from *A. palmata *and *M. faveolata *form stage-specific clusters (unigenes). From the total set of ESTs from each species, over 92% (Ap) and 97% (Mf) of clusters contain ESTs from a single life history stage.

Only a small number of unigenes are found in more than one library (i.e. they are stage specific) (Figure 2). We annotated the unigene set by assigning putative identities based on blastx hits to nr, swissprot and GO databases (Table 1). A significant portion of the library had no putative identities. Of the unigenes with no blast hits, we searched for open reading frames encoding at least 100 amino acids beginning with an ATG start codon. From 3,654 unigenes in *A. palmata*, we found 665 ORFs. From 1,732 unigenes identified from *M. faveolata*, we identified 160 ORFs. Since secreted proteins are likely targets in setting the stage for the initial host-symbiont cell contact, we searched for potential secretory signals of these unigenes using SignalP [30]. For *A. palmata *we identified 97 and 111 predicted signal peptides for *M. faveolata *we identified 22 and 28 predicted signal peptides based on 2 different methods (Hidden Markov Model and Neural Network algorithm), thus providing a starting set of genes of the "secretome" of the symbiosis.

### *A. palmata *and *M. faveolata *transcriptomes

**Figure 3:**
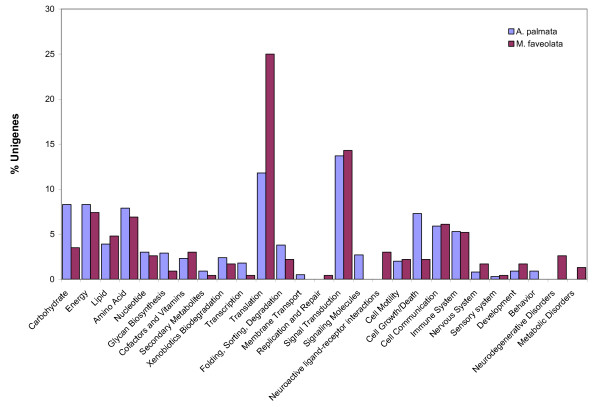
Large scale comparison of the transcriptomes of *Acropora palmata *and *Montastraea faveolata*. From the full set of unigenes identified for each species, KEGG assignments were possible for 659 unigenes of the 4980 unigenes identified from *A. palmata *and 230 unigenes of the 1732 identified for *M. faveolata*. Large scale differences between the transcriptomes of the two species occur in protein synthesis, carbohydrate metabolism and cell growth/death.

**Figure 4:**
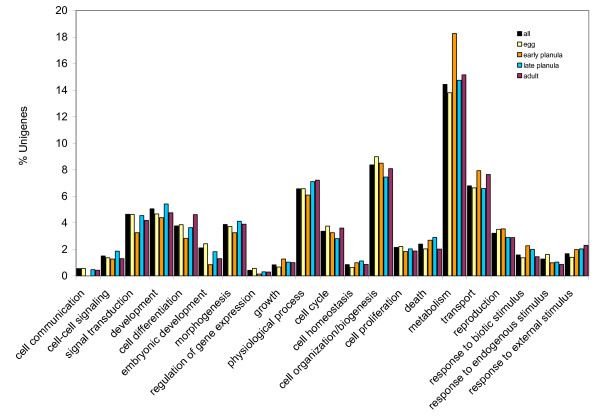
Large scale gene expression patterns throughout the life history of *A. palmata*. A total of 1295 unigenes were assigned Gene Ontology terms and summarized according to GO-defined biological processes and the life history stages in which those genes were expressed. Late planula larvae express a higher fraction of genes functioning in metabolism and transport, relative to the other life history stages.

Unigenes were categorized by biological processes using KAAS (KEGG Automatic Annotation Server). KEGG assignment could be made for 659 unigenes (13%) from *A. palmata *and 230 (10%) unigenes from *M. faveolata *(Figure 3). Large scale patterns of gene expression in the four major life-history stages that we sampled were compared using the dataset from *Acropora *(Figure 4), for which there was a sufficiently extensive EST dataset. Statistical analysis of the GO assignments for each unigene was performed using GeneMerge [31]. Genes expressed in each developmental stages were compared to the total set of genes from *A. palmata *to assess over-or under-representation of specific GO-defined categories in each developmental stage. For most developmental stages, there was no statistically significant difference, except for the library constructed from spawned eggs which showed significant over-representation of genes related to gene transcription, signal transduction, regulation of protein function, cell division and proliferation (Table 2).

**Table 2:** Over-representation of GO categories in unigenes from *A. palmata *eggs

Category	GO Term	Process	E-score	Description
DNA/RNA	GO:0030528	F	8E-03	Transcription regulator activity
	GO:0030528	C	8E-03	Small nuclear ribonucleoprotein complex
	GO:0005681	C	9E-06	Spliceosome complex
	GO:0031202	F	6E-04	RNA splicing factor activity, transesterification
	GO:0000398	P	7E-06	Nuclear mRNA splicing, via spliceosome
PROTEIN	GO:0004672	F	1E-02	Protein kinase activity
	GO:0004674	F	8E-07	Protein serine/threonine kinase activity
	GO:0004713	F	3E-05	Protein-tyrosine kinase activity
	GO:0007165	P	9E-06	Signal transduction
CELLULAR	GO:0008283	P	6E-03	Cell proliferation
	GO:0007067	P	1E-02	Mitosis
	GO:0000070	P	8E-03	Mitotic sister chromatid segregations
FUNCTION	GO:0003677	F	4E-07	DNA binding
	GO:0005515	F	1E-21	Protein binding
	GO:0005524	F	1E-03	ATP binding

### Identification of candidate genes

We characterized the unigene datasets by identifying those genes that were either expressed exclusively when the host was in the symbiotic stages (Table 3) or were expressed in all 5 sampled life history stages (Table 4). Five unigenes were expressed in all stages, while 15 unigenes are exclusively found in the symbiotic stages (i.e., found in both libraries LPI and AC). Seven of the 15 symbiosis-restricted unigenes could be assigned a putative identity based on blastx against nr, and these were biased in the category of DNA-interacting proteins (3 of 7). One EST-rich unigene (17cDNAs) could not be assigned a putative identity.

**Table 3:** Potential symbiosis-related genes in *A. palmata*

Unigene ID*	Length (nt)	#cDNAs	Blastx hit (nr)	e value
3742400	538	2	homothorax 1, homeodomain	10^-36^
3741518	411	3	chromobox homolog 1	10^-18^
3742103	376	2	THAP DNA binding domain	10^-5^
3740339	1444	3	aspartyl protease	10^-136^
3740760	793	3	leucine carboxyl methyltransferase 1	10^-59^
3741304	512	2	phosphoplipase A2 inhibitor	10^-3^
3740726	526	2	type III collagen	10^-13^
3742809	1601	17	no	
3743087	956	3	no	
3740331	862	3	no	
3741565	1147	2	no	
3740122	1045	2	no	
3742400	636	2	no	
3741088	585	2	no	
3743345	444	2	no	

* Criteria for inclusion were that the unigenes must be expressed in both symbiotic life history stages and absent from all of the non-symbiotic stages.

**Table 4:** Unigenes expressed in all life history stages of *A. palmata*

Unigene ID	Length (nt)	#cDNAs	Blastx hit (against nr)	e-value
3741124	1204	32	CCAAT/Enhancer binding protein	10^-15^
3741247	694	20	calmodulin	10^-75^
3741536	860	15	histone H3.3, Histone H3	10^-59^
3743425	1203	8	scotin pro-apoptotic protein	10^-7^
3740181	1416	17	No blastx or tblastx	

Using a candidate gene approach to identify potential host-symbiont cell-cell interaction proteins, we identified 25 potential pattern recognition receptors that are potentially involved in host-symbiont cell contact. For example, we identified a homolog of the horseshoe crab tachylectin-2, a protein which functions in the recognition of pathogens by *Tachypleus tridentatus *[32,33].

In addition, we identified partial sequences for signaling genes known to play roles in host response to microbial infection, for example members of the MAPK signaling pathway, NFkB signaling, and TIR domain protein. All of these signaling pathways play major roles in host responses to microbial infections. These genes are summarized in Table 5.

**Table 5:** Potential Pattern Recognition Receptors

Stage	Pfam ID	Accession	e-value	Swissprot (nr) top hit ID*	Accession	e-value
SE	Discoidin	PF00754.14	1.5E-25	CNTP2_PONPY Contactin-associated protein-like 2	73620138	3.0E-11
SE	Thrombospondin type 1	PF00090.8	1.8E-04	ATS18_HUMAN ADAMTS-18 precursor	76800647	2.0E-10
SE	Leucine Rich Repeat	PF00560.20	4.4E-17	LRC57_BRARE Leucine-rich repeat-containing protein	82235961	7.0E-63
SE	Leucine Rich Repeat	PF00560.20	4.8E-16	(PREDICTEDsimilar to scribble)	50769218	3.0E-70
SE	Leucine Rich Repeat	PF00560.20	8.5E-11	(PREDICTEDsimilar to hypothetical PRO1855)	55646569	1.0E-36
SE	Leucine Rich Repeat	PF00560.20	1.7E-11	RTN4R_MACFA Reticulon-4 receptor precursor	25453268	1.0E-14
EP	Scavenger receptor cysteine-rich	PF00530.8	8.5E-24	LOXL3_MOUSE Lysyl oxidase homolog 3	13878586	3.0E-21
EP	Scavenger receptor cysteine-rich	PF00530.8	4.8E-32	(similar to deleted in malignant brain tumors)	73998942	2.0E-30
LPU	Ricin-type beta-trefoil lectin	PF00652.11	1.5E-19	(hemolytic lectin CEL-III)	30060362	4.0E-98
LPU	Thrombospondin type 1	PF00090.8	1.6E-05	(Hypothetical protein F09F9.4)	17551566	8.0E-13
LPU	Scavenger receptor cysteine-rich	PF00530.8	1.2E-07	DMBT1_MOUSE Deleted in malignant brain tumors 1	85687557	8.0E-11
LPU	Chitin binding Peritrophin-A	PF01607.12	2.9E-07	(chitinase2)	46240806	6.0E-06
LPI	Scavenger receptor cysteine-rich	PF00530.8	3.1E-28	(similar to deleted in malignant brain tumors)	72138690	1.0E-24
LPI	Discoidin	PF00754.14	3.5E-30	MFGM_MOUSE Lactadherin precursor	46397820	9.0E-19
LPI	Thrombospondin type 1	PF00090.8	2.6E-07	HMCN1_HUMAN Hemicentin-1 precursor (Fibulin-6)	85542049	4.0E-12
LPI	Fibrinogen	PF00147.7	3.7E-72	FCN1_MOUSE Ficolin-1 precursor	13124179	2.0E-51
LPI	Putative peptidoglycan binding	PF01471.8	9.3E-04	(unnamed protein product)	4722818	3.0E-09
LPU LPI	Thrombospondin type 1	PF00090.8	7.1E-37	HMCN1_HUMAN Hemicentin-1 precursor (Fibulin-6)	85542049	3.0E-40
AC	Discoidin	PF00754.14	1.9E-11	FUCL_ANGAN Fucolectin	82131101	2.0E-17
AC	Thrombospondin type 1	PF00090.8	4.3E-22	SEM5A_MOUSE Semaphorin-5A precursor	8134718	1.0E-25
LPU	Leucine Rich Repeat	PF00560.20	7.5E-15	SHOC2_HUMAN Leucine-rich repeat protein SHOC-2	14423936	3.0E-59
LPI	Galactose binding lectin	PF02140.7	2.0E-06	(similar to latrophilin 2)	68400086	6.0E-08
AC	NA	NA	NA	TAL2_TACTR Tachylectin-2	18202523	1.0E-34

### Evolution of ferritin

**Figure 5:**
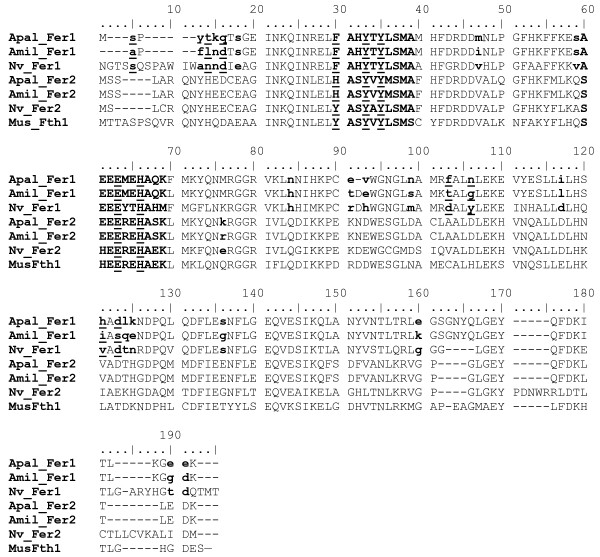
Amino acid alignments ferritin type I and type II from *A. palmata*, *A. millepora*, and *N. vectensis *with mouse H ferritin. Residues associated with the metal-binding site are written in *uppercase bold*. Tyr30, Tyr 33 and Tyr 35 that make up the ferroxidase center and Glu63 and His66 that play a role in polynuclear Fe-complex formation are *uppercase bold and underlined*. Tyr30 is specific for H ferritin in vertebrates [34,35]. Positively selected sites by a BEB procedure are *lowercase bold*, respectively *lowercase bold and underlined *if posterior probability of being in positively selected class > 0.5. Numbering is that of the mouse H chain sequence. Apal_Fer1: *A. palmata *ferritin type I, Amil_Fer1: *A. millepora *ferritin type I, Nv_Fer1: *N. vectensis *ferritin type I, Apal_Fer2: *A. palmata *ferritin type II, Amil_Fer2: *A. millepora *ferritin type II, Nv_Fer2: *N. vectensis *ferritin type II, MusFth1: mouse ferritin H chain.

We compared some of the most highly expressed genes in the *A. palmata *dataset with an EST dataset from *Acropora millepora*. Two different types of ferritins (referred to as ferritin type I and type II) were identified and that are highly expressed in both species. To differentiate the amino acids that are under positive selection, we identified homologues for both types of ferritin in the *Nematostella vectensis *database and carried out a site-specific analysis. Figure 5 illustrates alignments of the cnidarian ferritins to mouse ferritin, showing the amino acids predicted to be undergoing positive selection. The overall dN/dS value for the *A. palmata *and *A. millepora *EST sequence is ω = 1.0360 (dN= 0.0853, dS= 0.0823) for ferritin type I, and ω = 0.0799 (dN= 0.0093, dS= 0.1167) for ferritin type II. The dN/dS value for ferritin type I is unusually high and indicative of adaptive evolution. To differentiate the amino acids that are under positive selection, we identified homologues for both types of ferritin in the *Nematostella vectensis *database and carried out a site-specific analysis (Figure 5).

For ferritin type I, both tests give highly significant Likelihood Ratio Tests (LRTs) (M2a vs. M1a: p = 0.0003; M8 vs. M7: p = 0.0001) when analyzed for sites of positive selection. Seven sites are reported with a posterior probability of > 0.95 of being in the positively selected class. The Bayesian Empirical Bayes (BEB) procedure identified the same sites in Models M2a/M1a and M8/M7 but the M8/M7 test shows slightly higher probability values. In addition, one site is only identified in M8/M7 but with a low posterior probability (191E, Pr (ω > 1) = 0.67). For ferritin type II the LRTs are not significant. There is one site that is identified as positively selected in both tests but with a low probability (76 K, Pr (ω > 1) = 0.64).

Five highly conserved residues have been identified in vertebrate H chain ferritins. These are Tyr30, Tyr33, Tyr35 that make up the ferroxidase center and Glu63 and His66 that are involved in polynuclear Fe-complex formation (numbering based on the mouse H chain ferritin). All of these residues but Tyr30 are conserved in the coral ferritin type I molecules and type II molecules. Tyr30 is said to be specific for H ferritin in vertebrates [34,35]. In contrast, the *N. vectensis *ferritin type 2 sequence has this Tyr30 residue. None of the seven positively selected sites with a posterior probability of > 0.95 are in proximity of these domains.

### Novel genes restricted to scleractinian corals

**Figure 6:**
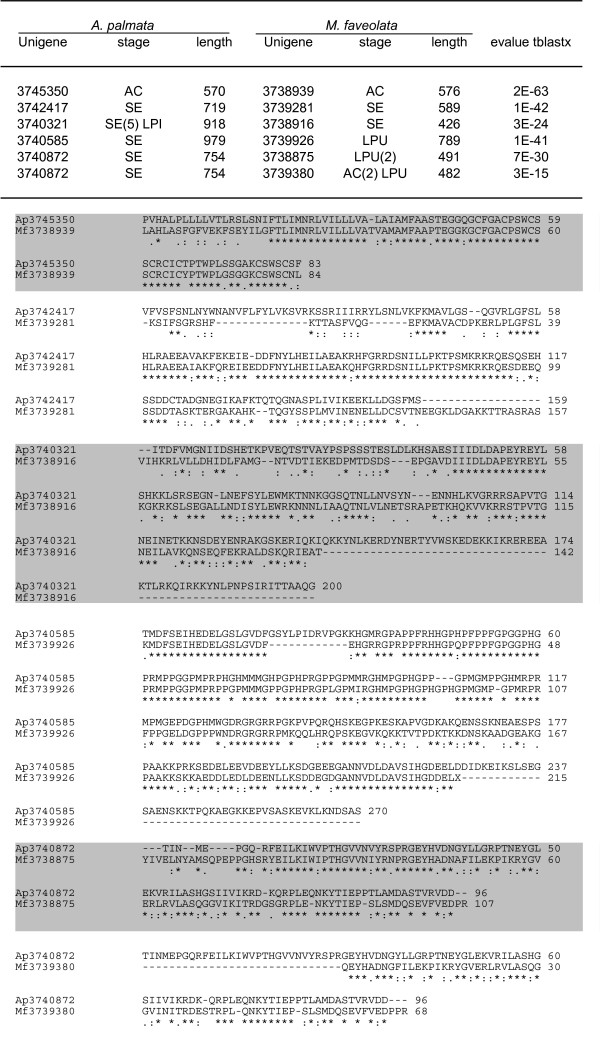
Novel genes restricted to corals. We identified 5 *A. palmata *genes and 6 *M. faveolata *unigenes that are highly conserved at the amino acid level and for which there are no matches to GenBank nr or nt at 1 × 10^-5^, and no matches to either *N. vectensis *or *H. magnipapillata *genomic sequences. Significance is shown as tblastx e-values and alignments for the open reading frames for each species. Notation in parentheses indicates the number of cDNAs present in each life history stage, if more than one.

To identify putative genes unique to scleractinian corals, we identified unigenes that were conserved between *A. palmata *and *M. faveolata *but that had no similarity to any sequences in nr, nor to the assembled genome of the sea anemone *Nematostella vectensis *or the assembled EST dataset from *Hydra magnipapillata*. We identified 5 genes in *A. palmata *that had matches to 6 genes in *M. faveolata*, but had no similarity to any sequences in nr nor to other cnidarian sequences. Amino acid alignments show that the majority of these predicted proteins are highly conserved (Figure 6). All but one of these unigenes contain ESTs from libraries prepared from non-symbiotic stages, strongly suggesting that these represent coral, and not symbiont, genes. The homologs *Ap: *3745350 and *Mf: *3738939, however are both from libraries constructed from adult coral tissue, suggesting that, given that a small percentage of ESTs from symbiotic stage were identified as symbiont genes, there is a small possibility that these homologs are from *Symbiodinium*.

## Discussion

Partial sequencing of 5 cDNA libraries for *A. palmata *and *M. faveolata *has produced a rich set of candidate genes (4,980 genes from *A. palmata*, and 1,732 genes from *M. faveolata*) that can be used as a starting point for examining the ecology and evolution of these two species from a cellular and molecular perspective. Toward these efforts, we have characterized: 1) the transcriptome across developmental stages, 2) unigenes that may play roles in symbiosis, and 3) candidate scleractinian-specific genes.

### *A. palmata *and *M. faveolata *transcriptome

There were very few genes that appeared to be ubiquitously expressed throughout the life history of either coral species. The evidence for this is that the *Acropora *EST dataset generated only 5 unigenes that were expressed in all 5 life history stages, and the *Montastraea *dataset produced none. In fact, the vast majority of unigene EST clusters are comprised of ESTs from a single developmental stage. This may reflect the conservative parameters of our clustering methods (to be clustered, reads must possess 96% identity across 100 or more nucleotides), but also is likely a consequence of sampling bias resulting from small sample size (small numbers of clones sequenced from each library). Only the spawned egg stage shows significant differences in the GO content in comparison to the overall EST collection. The egg is poised to initiate development of the zygote, thus it is expected to express at high levels genes involved in the first stages of cleavage and development, for example genes involved in cell proliferation, mitosis, regulation of transcription, and signal transduction. All of these categories were identified by Gene Merge as being over-represented in the egg stage.

### Candidate genes for initiation and regulation of the symbiosis

We used two approaches for identifying genes that may play roles in establishing or regulating the symbiosis. First we identified genes that are present in both symbiotic stages (*LPI, AC*) and absent in all non-symbiotic stages (*SE, EP, LPU*). Genes identified by this approach include DNA-binding proteins involved in regulating chromatin structure and transcription (hits to homothorax 1 homeodomain, chromobox homolog, and THAP DNA binding domain) that are potentially significant as regulators of gene expression during symbiosis. Identification of a potential homolog of leucine carboxylase methyltransferase may indicate regulation of protein phosphatase 2A, which is involved in control of many cellular functions, including metabolism, cell cycle progression, stress response, and apoptosis [36]. Although we can not rule out the possibility that some of the genes come from the symbiont, it is unlikely since all of the top blast hits were to animal genes, not plant or algal genes.

The cellular and molecular nature of the host response to microbial infections in cnidarians is an area of investigation that is rapidly being developed, and many of the well-studied components of the metazoan innate immune repertoire of pattern recognition and host response signaling pathways are present in cnidarians [16,37]. A small handful of players have been identified as playing roles in cnidarian-microbial interactions [21,38,39]. What is particularly interesting about studying innate immune response in corals is that a mutualistic infection of animals by eukaryotic microbes is known to occur only in a few marine invertebrate species, with corals being the poster child for this animal-microbe interaction. The ability to experimentally infect corals with their symbionts provides a study system both for characterizing the host response to microbial infection in basal metazoans, and for comparative studies of mutualistic vs. pathogenic infection in animals.

As a step forward, we report here the identification of genes containing protein domains that may play roles in mediating the initial host-symbiont contact, for example, lectin domains or other pattern recognition receptor domains that may be expressed on the host cell and interact with molecules on the symbiont cell surface. These included a diverse set of lectin domain, scavenger receptor domain, discoidin domain, ficolin, thrombospondin type 1 domain, Leucine rich repeat domain proteins, and others. We also identified candidate homologs to genes in signaling pathways known to be involved in host response to microbial infection, including members of the MAPK signaling pathway, Nuclear factor NF-kappa-B p105 subunit, and TIR domain signaling which functions as one of the major signaling pathways involved in response to microbial infection.

The identification of a homolog of the horseshoe crab protein tachylectin-2 is interesting, as this protein plays a significant role in the recognition of pathogens by the horseshoe crab, *Tachypleus tridentatus *[32]. In the hydrozoan cnidarian *Hydractinia*, the tachylectin homolog CTRN is structurally conserved, but is not upregulated after exposure to LPS, suggesting that it does not function as an immune response protein [40]. In *Hydractinia*, CTRN is expressed only in post-metamorphic stages, and in *Montastraea faveolata*, the tachylectin homolog was identified as a cDNA from the adult stages. The expression patterns of the *M. faveolata *tachylectin homolog throughout developmental and symbiosis should be examined to determine whether this gene appears to play a role in symbiosis.

### Sequence divergence in *Acropora*

The divergence between Caribbean and Indo-Pacific *Acropora *species is thought to substantially pre-date the closure of the Isthmus of Panama [41]and may be as deep as the mid Eocene (37–42 Ma) [42]. The *Acropora/Montastraea *divergence is much deeper, as the respective coral families were distinct in the Jurassic [43], and these two species belong to two divergent scleractinian clades that date back to at least the Triassic period (240–288 Ma) when corals appear to have originated [44].

Comparisons between the Caribbean and Indo-Pacific corals are complicated by the fact that full-length sequences are available for relatively few clones. Two of the largest EST clusters in the *A. millepora *dataset encode proteins matching strongly to invertebrate ferritins (Ball, Hayward et al., unpublished), and this is also true of both the *A. palmata *and *M. faveolata *datasets, however, relationships between these are not simple. The *Acropora *species each have orthologs of two distinct ferritin types, but the two *M. faveolata *EST clusters both correspond to one of these *Acropora *types. So as to avoid the complications of potentially comparing paralogs, the *M. faveolata *sequences were not subject to detailed comparative analysis.

One ferritin type (type I) is encoded by > 50 ESTs in the *Acropora millepora *dataset, and its ortholog in *A. palmata *is represented by 18 ESTs/11 cDNAs. The second ferritin type (type II) (45 ESTs in *A. millepora *and 10 in *A. palmata*) is more conserved. Type I is under positive selection but not type II. Type II ferritin has 9 synonymous and 4 non-synonymous nucleotide substitutions between the two *Acropora *species, whereas type I has 7 synonymous and 16 non-synonymous nucleotide changes.

It is intriguing that two different types of ferritins with different rates of evolution can be identified in *A. palmata*, *A. millepora *and *N. vectensis*. Ferritins play a major role in iron homeostasis and the oxidative stress response[45], they are important in innate immunity, for example, during the acute phase in response to pathogenetic infection [46,47]. For all of these reasons, it will be particularly interesting to examine the evolution and functions of ferritin in cnidarian biology and symbiosis.

### Genes restricted to the Scleractinia

The significance of candidate protein-coding genes that are clearly conserved between two divergent coral clades, yet absent from any other organism, is currently unknown. Three of these genes are expressed at the same life history stage of both species, suggesting that these proteins may play significant roles in co-ordinate life history stages in different coral species. The fourth gene is expressed in the adult colony of both species. The fifth gene is an *A. palmata *gene with two homologs in *M. faveolata*. Functional studies of these genes, and localization of the proteins will allow to further investigate the nature of scleractinian-specific genes (i.e. understanding how the genetic makeup of reef building corals differs from other cnidarians). In particular, they can potentially provide insightful knowledge on aspects of their evolutionary successful strategies such as biomineralization and photosynthetic symbiotic abilities.

## Conclusion

Partial sequencing of 5 cDNA libraries each for *A. palmata *and *M. faveolata *has produced a rich set of candidate genes (4,980 genes from *A. palmata*, and 1,732 genes from *M. faveolata*) from which we have identified many potential candidate genes for symbiosis regulation, ferritin genes that appears to be undergoing adaptive evolution, and five genes that may be restricted to Scleractinia. This EST resource can be used as a resource for examining the processed involved in the life history and symbiosis history of these two species, as well as to further expand the dataset of cnidarian genes for comparative genomics and evolutionary studies.

## Methods

### Collection of Material

Gametes from the two coral species were collected during mass spawning events in the Florida Keys at Horseshoe and Key Largo Dry Rocks (*Ap*) and Little Grecian (*Mf*) in August 2003, 2004 and 2005, as described in Szmant et al. [48]. Briefly, conical nets were suspended over spawning colonies to collect the positively buoyant gamete bundles. Gamete bundles from multiple colonies (and in the case of *A. palmata*, from multiple reefs) were combined within an hour to obtain cross-fertilization among different genets (these hermaphroditic species do not self-fertilize). Sperm concentrations were not measured but the gametes were kept concentrated in a ratio of 20 % gamete bundles to 80 % seawater to maintain high sperm concentrations. After one hour, sperm were washed out with several rinses of clean filtered seawater. Batches of fertilized eggs were put into 4 L plastic bins for culture at concentrations of about 2–3 thousand embryos per liter. Water was changed 2–3 times per day or more frequently if water quality conditions declined. *M. faveolata *larvae reached a swimming planula stage by 48 hours after fertilization, while those of *A. palmata *took about 60 hours.

### Infection studies

At 5 days post-fertilization for *M. faveolata *and 9 days post fertilization for *A. palmata*, larvae were randomly assigned to one of two parallel treatments: one to be infected with *Symbiodinium *and one to remain non-symbiotic. Larvae assigned to the infection treatment were inoculated with 1000 cells/ml of either strain Cass KB8 (Clade A, isolated from the jellyfish *Cassiopeia xamachana*) for *A. palmata*. *M. faveolata *larvae were inoculated with strains SSPe, Mf10.14b, and 704 (Clade B, from the gorgonian *Pseudopterogorgia elisabethae*, *M. faveolata*, and the gorgonian *Plexaura kuna*, respectively). Both species were maintained in the presence of *Symbiodinium *until sampled 6 days (*A. palmata*) or 8 days (*M. faveolata*) later. Larvae in both treatments were maintained at the same densities, were subjected to the same schedule of water changes, and were sampled at the same time. Upon sampling, larvae were washed to remove exogenous *Symbiodinium*, removed from the water, and snap frozen.

### cDNA library construction

We sampled tissue for construction of cDNA libraries from 5 different developmental/symbiotic life history stages for each species. For *Acropora palmata *we collected 1) freshly spawned eggs, 2) planula larvae at 96 hrs post-fertilization, 3) 15 day old larvae either infected with *Symbiodinium *ribosomal clade A strain Cass KB8 or 4) remaining uninfected, and 5) tissue from adult corals. For *Montastraea faveolata *we collected 1) freshly spawned eggs, 2) planula larvae at 60 hours post fertilization, 3) 13 day old larvae either infected with *Symbiodinium *ribosomal clade B strains SSPe, Mf10.146, and 704, or 4) remaining uninfected, and 5) tissue from an adult colony.

Total RNA was isolated from tissue samples using Qiazol reagent (Qiagen), according to manufacturer's instructions, and passaged through a 21G syringe to lyse the cells (larvae) or bombarded with glass beads to blast tissue off of the skeleton (adult colony). To remove residual phenol or other contaminants, the RNA was purified using an RNEasy clean up kit (Qiagen). Total RNA was quantified using a Nanodrop spectrophotometer, and RNA quality was assessed using an Agilent Bioanalyzer.

To construct the cDNA libraries, we used the Clontech SMART cDNA Library Construction Kit with the pDNR-lib vector. The cDNA was PCR-amplified using the Advantage 2 PCR kit, using the SMART 5' PCR III primer and CDS III/3' PCR primer, using between 18 and 26 cycles, depending on the starting amount of RNA. To minimize cloning incomplete or degraded transcripts, we preferentially selected cDNA > 500 bp, by first passing the *Sfi*I-digested cDNA over CHROMA SPIN-400 columns, and then in some cases, cutting out a > 500 bp smear from a 1.1% agarose gel. The size-selected cDNA was ligated to the pDNR-lib vector. Electrocompetant cells were transformed with the vector, grown overnight in liquid suspension and then plated onto Teknova LB agar plates with 30 μg/ml chloramphenicol. Colonies were picked into 384 well plates using a QBot robot (Genetix), and were sequenced from both 5' and 3 ' ends on ABI 3730 Sequencers at the Joint Genome Institute (JGI).

### EST Assembly and Generation of a Unigene dataset for each species

We generated a non-redundant set of genes from each of the two coral species, by grouping ESTs from all of the life history stages for each coral. For each species, EST clusters and consensus sequences were generated using the Joint Genome Institute's EST Analysis Pipeline, briefly described as follows. Base assignment and quality scores were assigned using the Phred software [49,50]. Vector, linker, adapter, poly-A/T, and other artifact sequences were removed using the Cross_match software (available with the Phrap package), and an internally developed short pattern finder. ESTs shorter than 100 bp were removed from the data set, as were contaminant sequences, such as *E. coli*, common vectors, and sequencing standards. To enrich for nuclear protein-coding genes, we removed rRNA and mitochondrial DNA sequences from the EST dataset prior to alignment and clustering.

Pair-wise EST alignments were generated using the Malign software (Chapman, et. al., unpublished), a modified version of the Smith-Waterman algorithm [51] which was developed at the JGI for use in whole genome shotgun assembly. ESTs sharing an alignment of at least 96% identity, and 100 bp overlap are assigned to the same cluster. ESTs from the same cDNA were assigned to the same cluster even if they did not overlap. Consensus sequences for the EST clusters (unigenes) were generated using Cap3 [52]. The resulting EST and unigene datasets is generally free from vector, *E. coli*, coral rRNA and mtDNA sequences, and therefore represents a predicted set of nuclear protein-coding genes and non-classified RNAs.

ESTs described in this paper are available through NCBI: GenBank accession DR982333–DR988505, EY021828–EY031784 and FE038910–FE040597.

### Identification of Symbiont ESTs in the host unigene sets

To estimate the extent of contamination of host libraries from symbiont RNA, we performed a tblastx search of the coral unigenes against a *Symbiodinium *sequence database, consisting of 2002 nucleotide sequences available in GenBank for the genus *Symbiodnium*. Genes that were identified from this process were then checked by tblastx against nr to confirm that *Symbiodinium *or another dinoflagellate or plant was the best hit. Such genes were concluded to be contaminants from the symbiont. We identified only 1 cDNA in *M. faveolata*, which was clearly from *Symbiodinium *(top tblastx hit to a *Symbiodinium cob*). In *A. palmata*, we detected 3 protein coding genes from *Symbiodinium*: 4 cDNAs encoding peridinin chlorophyll protein (the major accessory pigment protein in *Symbiodinium*), 4 cDNAs encoding *cob *from *Symbiodinium*, and 1 cDNA encoding *cox1 *(top hit was to another dinoflagellate, *Pfiesteria piscicida*).

### Homology searching and prediction of protein-coding genes and secreted proteins

To functionally characterize our unigene sets, we performed a blastx analysis (e-value cutoff 1e^-5^) against three databases: the GenBank non-redundant DNA and protein databases (nr), the Swiss-Prot database of manually curated protein sequences (swissprot), and the Gene Ontology database of controlled vocabulary terms that describe gene and protein attributes.

As only a subset of the unigenes returned significant hits to any of these databases, we took other approaches to identify unigenes that might function in the establishment and maintenance of the symbiosis. (1) We identified protein-coding ESTs from the set of unigenes that had had no blast hits, using the GETORF algorithm. Using this algorithm, we identified unigenes that contained at least a 300 nt open reading frame beginning with the ATG start codon. (2) We used SignalP to identify the canonical N-terminal amino acid motif that targets nascent proteins into the classical ER-secretory pathway[30]. Secreted proteins and membrane-associated proteins are known from other systems to play a significant role in host-pathogen interactions, particularly in the initial recognition process, and are therefore of particular interest for studying the coral-*Symbiodinium *interaction.

### *A. palmata *and *M. faveolata *transcriptomes

To compare the transcriptomes between the two species, we compared the *A. palmata *unigene dataset to the *M. faveolata *dataset, using KEGG-assigned categories [53], through submission of the unigene datasets to the KAAS web-based annotation tool. This method generates BLAST comparisons against the KEGG GENES database, to assign KEGG Orthology identities. Genes were summed to represent larger-order biological processes. To examine large scale differences in gene expression between life history stages, we used the more extensive dataset from *A. palmata *to compare large-scale patterns of gene expression in the four major life history stages that we sampled (egg, early planula stage, late planula stage, and adult). We used the Gene Ontology assignments to classify the libraries by GO-defined Biological Process and Gene Ontology Terms which were mapped to larger order biological processes.

We then identified developmental stages that contained a significantly higher or lower number of genes functioning in each biological process, using GeneMerge, a statistical tool for generating rank scores for over-representation in each study set of genes (each developmental stage), by comparing it to the whole population of unigenes (all developmental stages) [31].

### Prediction of candidate symbiosis genes

We searched for genes that may be functioning in regulating symbiotic interactions between the host and symbiont by examining EST datasets for those unigenes that were expressed in the two symbiotic stages that we sampled (LPI, AC), and were not expressed in any of the stages that lack symbionts (E, P, LPU) (See Table 1 for abbreviations). We cannot exclude the possibility that some of the putative symbiosis-related ESTs are actually from the symbiont, although there was no evidence from the blast results to support this, as the top BLAST hits were to animal genes.

Comparisons of the cellular and molecular basis for pathogenic vs. mutualistic animal-bacterial associations are revealing that host-bacterial interactions are structured similarly regardless of whether the interaction is mutualistic or pathogenic[54]. As a result, it is extremely useful as a starting point to identify components of the innate immune and host response systems that have been identified from pathogenic or parasitic associations. The coral-dinoflagellate symbiosis adds a new dimension to understanding host-microbial associations, as the coral microbial partner is a eukaryote (related to the apicomplexan parasites such as *Plasmodium *and *Toxoplasma*), rather than a prokaryote. To identify candidate genes that may function in the host-microbe interaction processes, we examined our EST datasets to identify 1) protein domains (search against Pfam database of protein domains) and genes (blastx against nr and Swissprot) that are known to play roles in 1) cell-cell or pattern recognition interactions, and 2) signaling pathways that are known to be involved in host response to microbial infection.

### Comparisons between cnidarian datasets

To identify potentially significant genes involved in the evolution of corals, we performed analyses to identify genes in common to scleractinians that appear to be under positive selection. Ferritin type I corresponds to DR984234 in *A. palmata *and DY579151 in *A. millepora*. Ferritin type II corresponds to EST accession DR985990 in *A. palmata *and DY577778 in *A. millepora*. A tblastx of *A. palmata *against *Nematostella *ESTs identified homologues of ferritin type I and type II in *N. vectensis*. The best tblastx matches that are in the NCBI nucleotide database were used (*N. vectensis *ferritin type I: gi|82866539|, E-value 4e-57; *N. vectensis *ferritin type II: gi|82875723|, E-value 9e-77).

We tested for evidence of positive selection by comparing the nonsynonymous substitution rate (dN) to the synonymous substitution rate (dS). We used site-specific Maximum Likelihood models (ML) to detect positive selection on specific amino acids. We implemented models M1a (neutral) and M2a (selection) and M7 (beta) and M8 (beta&ε) [55-57]with the codeml program in PAML [58]. Alignment gaps and ambiguity characters are removed in PAML prior to dN/dS calculations. Data analyses and computer simulations have showed that these pairs of site models are well suited to detect positively selected sites [56,59-61]. A likelihood-ratio test (LRT) was used to compare the neutral with the corresponding selection models. The test statistic -2ΔlnL follows a χ^2 ^distribution with critical values to be 5.99 and 9.21 at 5% and 1% (*df *= 2). When the LRT is significant a Bayes Empirical Bayes (BEB) procedure was used to identify amino acid under positive selection. Overall dN/dS values ('one-ratio' model) were calculated with the Model M0. The F3x4 model of codon frequencies was used.

To identify genes that may be restricted to scleractinian corals, we identified unigenes that were highly similar between *A. palmata *and *M. faveolata *but that had no similarity to any sequences in nr, nor to the assembled genome of the sea anemone *Nematostella vectensis *or the assembled EST dataset from the hydrozoan *Hydra magnipapillata*.

## Authors' contributions

MM, AS, MAC, and JS carried out the sampling and infection experiments. JS made the cDNA libraries, oversaw the sequencing, participated in the assembly and annotation of the EST datasets, analyzed the EST datasets, and drafted the manuscript. PB assembled and PB, JS, and AT annotated the EST datasets. DM participated in the comparative analysis. CV carried out parts of the molecular evolution analysis. All authors read, edited, and approved the final manuscript. Cass KB8 culture originally isolated by Robert Kinzie III, Univ. of Hawaii.
