# Coral life history and symbiosis: Functional genomic resources for two reef building Caribbean corals, *Acropora palmata *and *Montastraea faveolata*

**DOI:** 10.1186/1471-2164-9-435

**Published:** 2008-09-23

**Authors:** Jodi A Schwarz, Peter B Brokstein, Christian Voolstra, Astrid Y Terry, Chitra F Manohar, David J Miller, Alina M Szmant, Mary Alice Coffroth, Mónica Medina

**Affiliations:** 1 Joint Genome Institute, Department of Energy, 2800 Mitchell Drive, Walnut Creek, CA 94598, USA; 2 School of Natural Sciences, PO Box 2039, University of California, Merced, CA 95344, USA; 3 Celera, 1300 Harbor Bay Parkway, Alameda CA 94502, USA; 4 Comparative Genomics Center, Molecular Sciences Building 21, James Cook University, Townsville, Queensland 4811, Australia; 5 Center for Marine Science, Center for Marine Science, 5600 Marvin K. Moss Lane, Wilmington, NC 28409, USA; 6 Department of Geological Sciences, 661 Hochstetter Hall, State University of New York, Buffalo, NY 14260, USA

## Correction

After the publication of this manuscript [1], a collaborator on initial phases of the project requested inclusion on the author list. We agree with that assessment. The author has been added to the author list and her contributions are listed in the Authors' Contributions section.

## Authors' contributions

MM, AS, MAC, and JS carried out the sampling and infection experiments. JS made the cDNA libraries, oversaw the sequencing, participated in the assembly and annotation of the EST datasets, analyzed the EST datasets, and drafted the manuscript. PB assembled and PB, JS, and AT annotated the EST datasets. CFM helped in the field and in optimization of the RNA extraction protocol. DM participated in the comparative analysis. CV carried out parts of the molecular evolution analysis. All authors read, edited, and approved the final manuscript. Cass KB8 culture originally isolated by Robert Kinzie III, Univ. of Hawaii.

## References

[B1] Schwarz JA, Brokstein PB, Voolstra C, Terry AY, Miller DJ, Szmant AM, Coffroth MA, Medina M (2008). Coral life history and symbiosis: functional genomic resources for two reef building Caribbean corals, *Acropora palmata *and *Montastraea faveolata*. BMC Genomics.

